# Frequent and Efficient Use of the Sister Chromatid for DNA Double-Strand Break Repair during Budding Yeast Meiosis

**DOI:** 10.1371/journal.pbio.1000520

**Published:** 2010-10-19

**Authors:** Tamara Goldfarb, Michael Lichten

**Affiliations:** Laboratory of Biochemistry and Molecular Biology, Center for Cancer Research, National Cancer Institute, Bethesda, Maryland, United States of America; Stowers Institute for Medical Research, United States of America

## Abstract

Recombination between homologous chromosomes of different parental origin (homologs) is necessary for their accurate segregation during meiosis. It has been suggested that meiotic inter-homolog recombination is promoted by a barrier to inter-sister-chromatid recombination, imposed by meiosis-specific components of the chromosome axis. Consistent with this, measures of Holliday junction–containing recombination intermediates (joint molecules [JMs]) show a strong bias towards inter-homolog and against inter-sister JMs. However, recombination between sister chromatids also has an important role in meiosis. The genomes of diploid organisms in natural populations are highly polymorphic for insertions and deletions, and meiotic double-strand breaks (DSBs) that form within such polymorphic regions must be repaired by inter-sister recombination. Efforts to study inter-sister recombination during meiosis, in particular to determine recombination frequencies and mechanisms, have been constrained by the inability to monitor the products of inter-sister recombination. We present here molecular-level studies of inter-sister recombination during budding yeast meiosis. We examined events initiated by DSBs in regions that lack corresponding sequences on the homolog, and show that these DSBs are efficiently repaired by inter-sister recombination. This occurs with the same timing as inter-homolog recombination, but with reduced (2- to 3-fold) yields of JMs. Loss of the meiotic-chromosome-axis-associated kinase Mek1 accelerates inter-sister DSB repair and markedly increases inter-sister JM frequencies. Furthermore, inter-sister JMs formed in *mek1*Δ mutants are preferentially lost, while inter-homolog JMs are maintained. These findings indicate that inter-sister recombination occurs frequently during budding yeast meiosis, with the possibility that up to one-third of all recombination events occur between sister chromatids. We suggest that a Mek1-dependent reduction in the rate of inter-sister repair, combined with the destabilization of inter-sister JMs, promotes inter-homolog recombination while retaining the capacity for inter-sister recombination when inter-homolog recombination is not possible.

## Abstract 2

Studies of DNA double-strand break repair during meiosis reveal that a substantial fraction of recombination occurs between sister chromatids.

## Author Summary

In diploid organisms, which contain two parental sets of chromosomes, double-stranded breaks in DNA can be repaired by recombination, either with a copy of the chromosome produced by replication (the sister chromatid), or with either chromatid of the other parental chromosome (the homolog). During meiosis, recombination with the homolog ensures faithful segregation of chromosomes to gametes (sperm or egg). It has been suggested that use of the spatially distant homolog, as opposed to the nearby sister chromatid, results from a meiosis-specific barrier to recombination between sister chromatids. However, there are situations where meiotic recombination must occur between sister chromatids, such as when recombination initiates in sequences that are absent from the homolog. By studying such a situation, we show that meiotic recombination with the sister chromatid occurs with similar timing and efficiency as recombination with the homolog. Further analysis indicates that inter-sister recombination is more common than was previously thought, although still far less prevalent than in somatic cells, where inter-sister recombination predominates. We suggest that meiosis-specific factors act to roughly equalize repair from the sister and homolog, which both allows the establishment of physical connections between homologs and ensures timely repair of breaks incurred in regions lacking corresponding sequences on the homolog.

## Introduction

During meiosis, the diploid genome is reduced to produce haploid gametes through two successive rounds of nuclear division that follow a single round of DNA replication. Homologous parental chromosomes (homologs) pair and separate at the first meiotic division (MI), while sister chromatids segregate during the second division. Crossover (CO) products of inter-homolog (IH) recombination, combined with sister chromatid cohesion, ensure proper chromosome disjunction at MI, and a failure to properly create these connections results in aneuploid progeny. Aneuploidy caused by MI non-disjunction is a leading cause of both miscarriage and congenital birth defects [1].

Meiotic recombination is initiated by double-strand breaks (DSBs) formed by the Spo11 protein [2]. DSBs are resected to form single strands that are substrates for strand invasion catalyzed by the meiosis-specific Dmc1 and the ubiquitous Rad51 proteins [3]. The choice of a target for strand invasion and subsequent repair during meiosis is distinct from that during the mitotic cell cycle. During the mitotic cell cycle, there is a strong bias to repair DSBs using the sister chromatid [4],[5]. In contrast, the homolog is often used to repair DSBs during meiosis, with two possible outcomes. After initial repair synthesis, the invading strand can detach from the homolog and reanneal with the unresected strand of the second DSB end to form a noncrossover (NCO) product in a process called synthesis-dependent strand annealing [6]. Alternatively, if the second end of the DSB also associates with donor sequences, Holliday junction–containing intermediates, here called joint molecules (JMs), are formed [7]. In budding yeast, these are mostly resolved as COs [8],[9].

It is generally thought that IH recombination dominates during meiosis. In budding yeast, most JMs form between parental homologs, with only 13%–25% forming between sister chromatids [7],[10]–[12]. *dmc1* mutants fail to repair DSBs and do not form JMs during meiosis, but rapidly form inter-sister (IS) JMs, and not IH JMs, when returned to vegetative growth [10]. In haploid yeast undergoing meiosis, a large fraction of DSBs persist unrepaired, suggesting that IS DSB repair is inefficient [13],[14]. These findings have been taken as evidence for a meiosis-specific barrier to sister chromatid recombination (BSCR) that prevents IS recombination and thus promotes IH recombination.

The axial element is a structure that forms between sister chromatids early in meiotic prophase. It later becomes part of the synaptonemal complex, a tripartite structure with axes of each homolog closely juxtaposed by transverse filaments [15]. In budding yeast, axial element components Red1 and Hop1, along with the axis-associated, meiosis-specific Mre4/Mek1 kinase (hereafter Mek1), have been suggested as mediating a BSCR [16],[17]. Recent studies indicate that meiotic DSBs activate the Mec1 and Tel1 checkpoint kinases, which phosphorylate Hop1 [17],[18]. Phosphorylated Hop1 binds and activates the Mek1 kinase, which phosphorylates targets that include the Rad51 accessory factors Rad54 and Rdh54 [19],[20]. This prevents interactions between these factors and Rad51 and thus is thought to decrease IS recombination.

Evidence consistent with this mechanism is provided by several findings. While DSBs accumulate to normal levels in DSB processing/repair-defective *mek1* *rad50S* double mutants [21],[22], *mek1* single mutants display reduced steady-state DSB levels and reduced IH COs [21],[23], as would be expected if DSBs were rapidly repaired by IS recombination in the absence of axis-mediated signaling. Consistent with this, both *red1* and *mek1* mutants display a marked excess of IS JMs over IH JMs [10],[24]. Further support for the suggestion that loss of axis signaling allows rapid IS recombination comes from findings that the DSB repair defect of *dmc1* mutants is suppressed by *hop1*, *red1*, or *mek1* loss of function mutations [10],[17],[19]–[21],[25], and that *mek1* suppresses the DSB repair defect seen in haploid yeast undergoing meiosis [14]. Additionally, the meiotic repair defect of *dmc1* mutants is partially suppressed by overexpression of *RAD51* [26] or *RAD54* [25], and more extensively by overexpression of a *RAD54* allele that lacks a Mek1 phosphorylation site [20].

These findings, while consistent with a Mek1-dependent BSCR during meiosis, were obtained under circumstances where repair and recombination are altered genome-wide. In particular, abnormally high levels of unrepaired DSBs in *dmc1* mutants and in haploid cells undergoing meiosis may result in altered repair mechanisms and outcomes. For example, the resection and repair of meiotic DSBs formed by the site-specific VDE endonuclease are altered in *dmc1* mutants by the presence or absence of other hyper-resected Spo11-catalyzed DSBs [27],[28].

While it is clear that IS recombination is less prevalent during meiosis than during vegetative growth, knowledge of the relative efficiency of IH and IS recombination during meiosis remains incomplete. Previous studies have inferred the relative frequency of IS and IH repair by comparing IS- and IH-containing JM intermediates. However, no study has directly measured the efficiency of all types of IS repair in normal diploids, partly because such measurements are hampered by the inability to detect many of the products of IS recombination. To address this issue, we monitored the fate of a DSB that could only be repaired by sister chromatid recombination, in cells where all other DSBs could be repaired by IH recombination. We show here that during normal diploid meiosis, such DSBs are efficiently repaired from the sister chromatid. This IS repair has many of the features of normal IH recombination, except that fewer JM intermediates are produced. Based on these and other observations, we suggest that repair from the sister occurs frequently during budding yeast meiosis, even when the homolog is present. We propose that the apparent BSCR is actually a kinetic impediment, imposed by the Mek1 kinase, that roughly equalizes rates of IS and IH recombination during meiosis, a process that would otherwise greatly favor IS events given the spatial proximity of the sister chromatid.

## Results

### Meiotic DSBs Are Efficiently Repaired in the Absence of Corresponding Sequences on the Homolog

**Figure 1:**
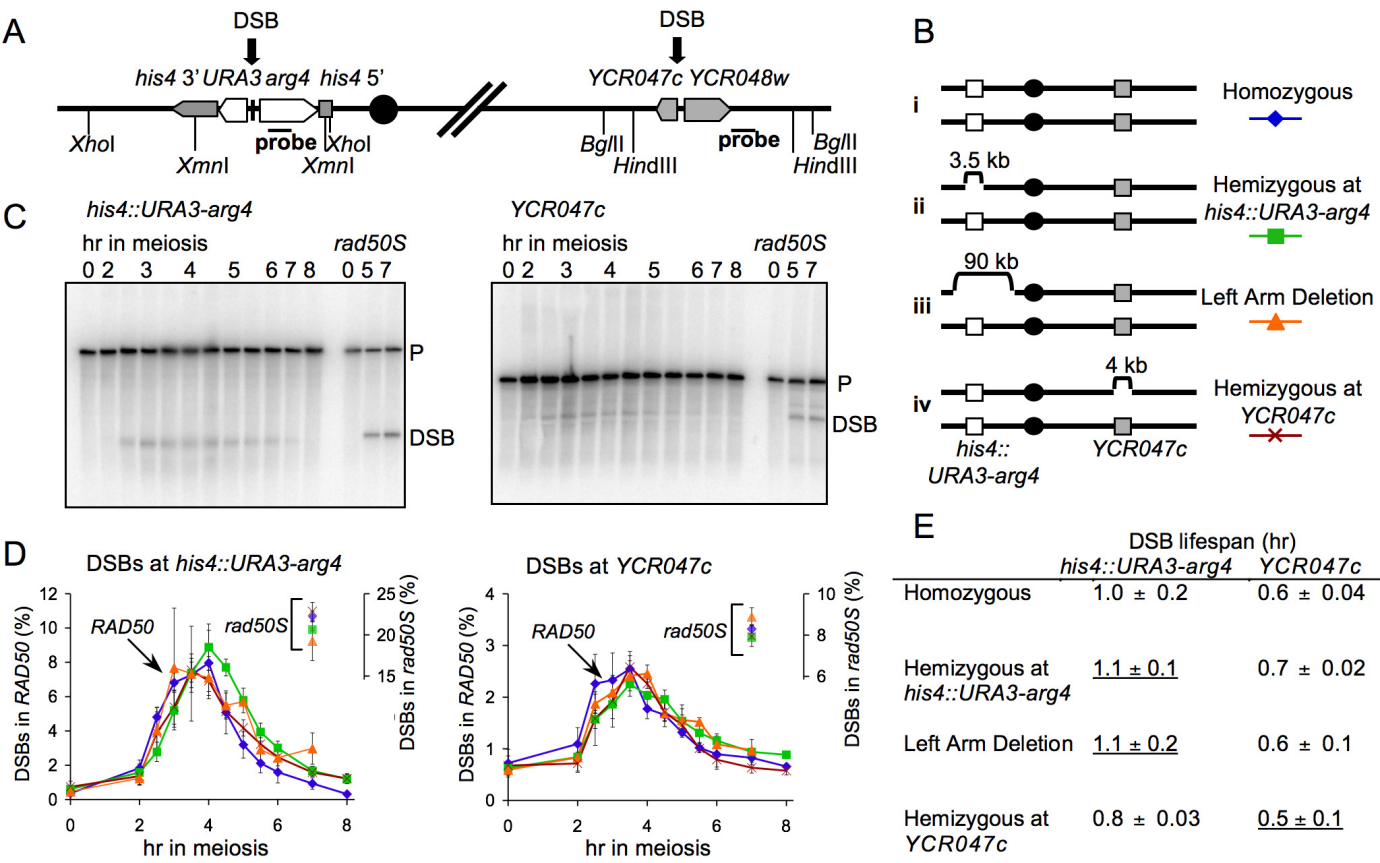
Similar timing of IS and IH DSB repair. (A) Structure of DSB hotspots in a 3.5-kb *his4::URA3-arg4* insert [29] and in the *YCR047c–YCR048w* intergenic region [82]. White boxes indicate *URA3-arg4* insert genes; grey boxes indicate other genes on chromosome *III*; horizontal bars indicate sequences used for probes [8],[29]; vertical lines indicate restriction sites used to detect DSBs (short lines) and JM intermediates (long lines). (B) Strains used. (i) Homozygous control—*his4::URA3-arg4* and *YCR047c–YCR048w* are present on both homologs. (ii) Hemizygous at *his4::URA3-arg4*—the 3.5-kb *his4::URA3-arg4* insert is present on only one homolog. (iii) Left arm deletion—the *his4::URA3-arg4* insert is present on one homolog, opposite a 90-kb deletion on the other homolog. (iv) Hemizygous at *YCR047c*—4 kb of DNA between *YCR046c* and *YCR051w* is deleted from one homolog. (C) Southern blots showing detection of DSBs in wild-type and *rad50S* strains hemizygous for *his4::URA3-arg4* at either the *his4::URA3-arg4* site (left) or the *YCR047c* homozygous control site (right). P, parental band; DSB, DSB band. (D) DSB frequencies (3–4 independent experiments, error bars indicate SEM), quantified as percent of total lane signal. Symbols connected by lines are noncumulative DSB frequencies from *RAD50* strains (left-hand *y*-axis); unconnected symbols at 7 h are cumulative DSB frequencies from *rad50S* strains are (right-hand *y*-axis). (E) DSB life span, calculated using 7-h *rad50S* cumulative DSB levels, as described previously [81]. Underlined life spans are for DSBs that must be repaired by IS recombination. Strains in (C–E) are (for *RAD50* and *rad50S*, respectively): homozygous, MJL3201 and MJL3198; hemizygous at *his4::URA3-arg4*, MJL3250 and MJL3338; left arm deletion, MJL3227 and MJL3233; and hemizygous at *YCR047c*, MJL3399 and MJL3408.

We examined DSBs at two hotspots on chromosome *III*: within a 3.5-kb recombination reporter construct containing *URA3* and *ARG4* sequences inserted at *HIS4* (*his4::URA3-arg4*; [29]) and in the *YCR047c* promoter (Figure 1A). Both DSB hotspots were examined in a hemizygous configuration, where the hotspot was present on one copy of chromosome *III* and a small deletion was present on the other homolog. This eliminates the possibility of repair of the hotspot DSB by IH recombination (Figure 1B), but preserves normal homolog alignment, synapsis, and IH recombination in the genome as a whole. We also examined a strain hemizygous for a deletion that removes most of the chromosome *III* left arm, including sequences for about 45 kb to either side of the *his4::URA3-arg4* insertion site (Figure 1B). In most experiments, DSB dynamics were examined in the same strain at a hemizygous site and at a homozygous control site, to control for culture-to-culture variation in meiotic progression and the fraction of cells undergoing meiosis.

It has been reported that heterozygosity for a small deletion covering a DSB site causes a modest reduction in DSB levels [30],[31]. This is not the case for the loci and deletions used here. Cumulative DSB levels at both the *his4::URA3-arg4* insert and *YCR047c* were measured in *rad50S* mutants, which form, but do not repair, DSBs [32]. DSBs accumulated to similar levels in deletion hemizygotes and in homozygous controls (Figure 1D, right-hand axes). In *RAD50* strains, similar DSB dynamics were seen when corresponding sequences were present on or absent from the homolog (Figures 1D and S1). Calculated DSB life spans (Figure 1E; see Materials and Methods) at both loci were similar in the presence or absence of homology on the homolog. To confirm that the absence of corresponding homology at one DSB site did not have a chromosome-wide effect on repair, DSB levels were also examined at a DSB site (*YFL021w*) on chromosome *VI*. Similar noncumulative DSB curves were observed at this site and at the tester sites on chromosome *III* (Figure S1D; data not shown).

**Figure 2:**
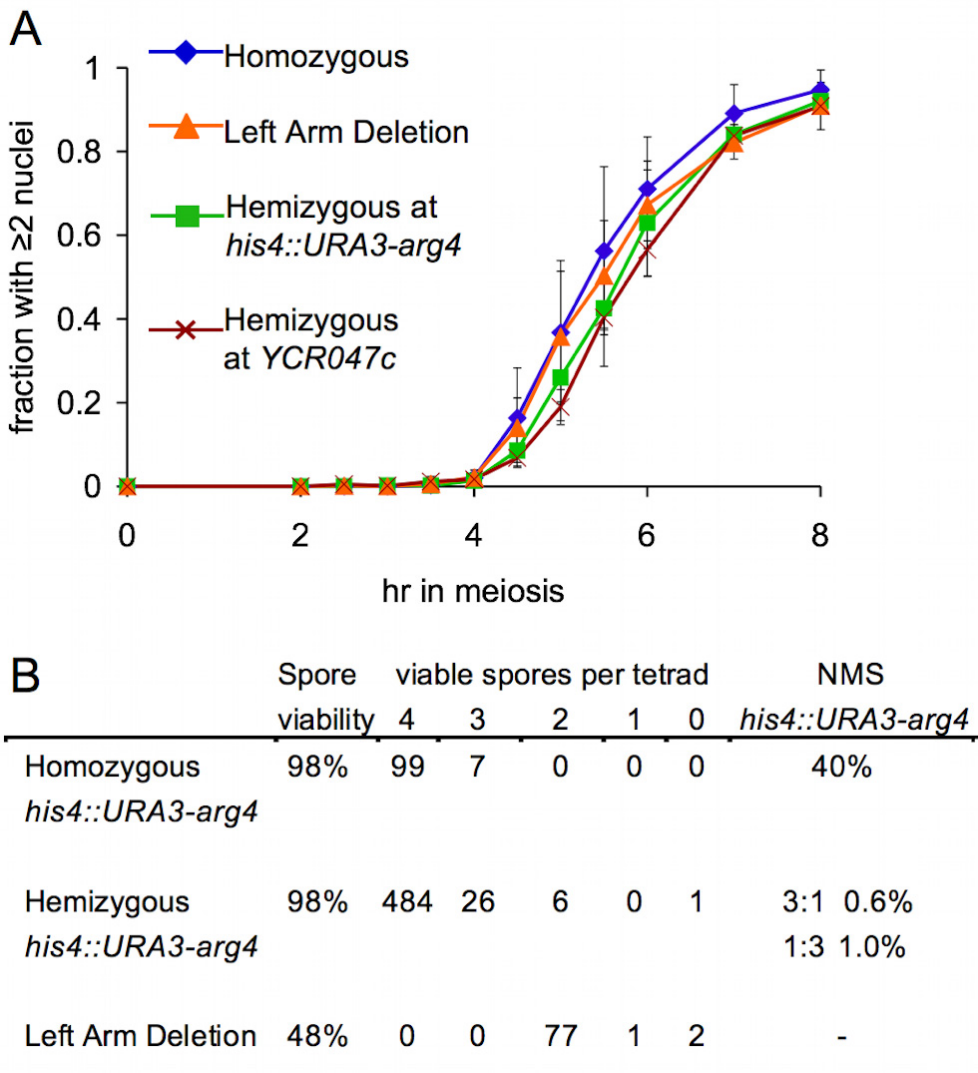
DSBs at a hemizygous locus do not alter nuclear division timing or spore viability. (A) Timing of the meiosis I nuclear division, monitored by DAPI staining (see Protocol S1). Values are the average of 3–4 experiments (error bars indicate standard deviation). *RAD50* strains as in Figure 1. (B) Spore viability in tetrads in the indicated strains. NMS indicates non-Mendelian segregation (full conversion and post-meiotic segregation) at the *his4::URA3-arg4* insert. The strain homozygous for the insert (MJL3195) is a *his4::URA3-arg4-pal/his4::ura3-pal-ARG4* trans-heterozygote. Non-Mendelian segregation at *ura3-pal* and at *arg4-pal* were scored; non-Mendelian segregation for both markers in the same tetrad was scored as a single event. In the *his4::URA3-arg4* hemizygote (MJL3192), non-Mendelian segregation events involved loss (1∶3) or gain (3∶1) of the *URA3-arg4* insert.

A BSCR-induced delay in DSB repair at a hemizygous site might cause a DNA-damage-response-induced delay in the MI division. We did not observe a significant difference between *his4::URA3-arg4* or *YCR047c* hemizygotes and fully homozygous controls for meiotic division timing or for the fraction of cells that transited meiotic divisions (Figure 2A; data not shown). In addition, no loss of spore viability was observed in strains hemizygous for the *his4::URA3-arg4* insert (Figure 2B), as might be expected if an unrepaired DSB persisted through meiotic divisions and sporulation.

While these findings are consistent with efficient repair of a DSB in hemizygous sequences by IS recombination, DSB end resection past the region of heterology and subsequent strand invasion of the homolog could also result in repair, by IH gene conversion, leading to loss of the hemizygous sequences. Given DSB frequencies at *his4::URA3-arg4* (about 20% of insert-bearing chromosomes), DSB repair by IH gene conversion would result in 36%–40% of tetrads showing 1∶3 segregation for the insert. However, only 1% of tetrads from a *his4::URA3-arg4* hemizygote showed this segregation pattern (Figure 2B).

In summary, all available molecular, meiotic progression, and spore survival data indicate that, when no other homologous repair partners are available at a DSB site, the sister chromatid is used as efficiently for meiotic DSB repair as would be the homolog—at least in strains where most other DSB sites are homozygous and can be repaired by IH recombination.

### Reduced JM Formation in the Absence of Corresponding Sequences on the Homolog

Synthesis-dependent strand annealing, which does not involve Holliday junction–containing intermediates, is thought to be the predominant mechanism of DSB repair during the S and G2 phases of the mitotic cell cycle [5],[33],[34] and for NCO formation during meiosis [8],[35],[36]. To test the possibility that synthesis-dependent strand annealing predominates during meiotic IS DSB repair, we asked whether meiotic JMs formed at a hemizygous locus. Such JMs must be IS recombination intermediates.

**Figure 3:**
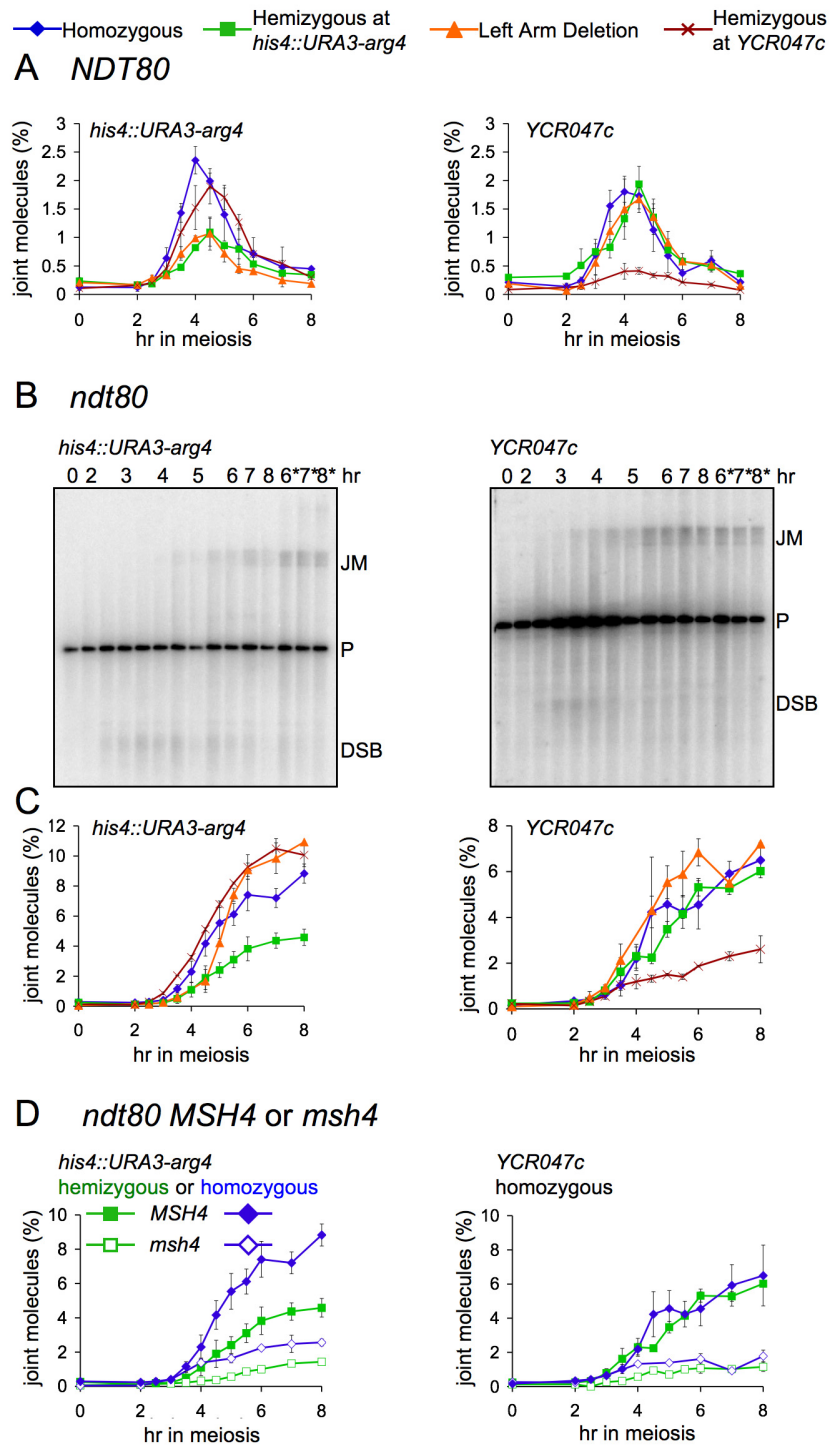
Reduced JM formation during IS chromatid recombination. (A) Noncumulative JM levels in wild-type strains at the *his4::URA3-arg4* insert and at *YCR047c*, expressed as percent of total signal in the lane. Each line represents the average of 3–4 experiments (error bars indicate SEM). (B) Southern blots of DNA from *ndt80*Δ strains hemizygous for *his4::URA3-arg4* (MJL3497) or homozygous for *his4::URA3-arg4* (MJL3252, denoted by asterisks) detecting JM intermediates at either *his4::URA3-arg4* (left) or at the homozygous *YCR047c* site (right). P, parental. (C) JM frequencies in *ndt80*Δ strains, quantified as percent of total lane signal. Each point represents the average of 2–4 experiments (error bars indicate SEM). Strains used: fully homozygous, MJL3252, blue diamonds; hemizygous at *his4::URA3-arg4*, MJL3497, green squares; 90-kb left arm deletion, MJL3245, orange triangles; hemizygous at *YCR047c–YCR048w*, MJL3406, red Xs. (D) IS JM formation is Msh4-dependent. JM frequencies (error bars indicate SEM), quantified as percent of total lane signal, in DNA from *ndt80*Δ strains hemizygous (green) or homozygous (blue) for *his4::URA3-arg4* and either *MSH4* (MJL3497 or MJL3252, filled symbols) or *msh4*Δ (MJL3385 or MJL3386, open symbols). Each point represents the average of 2–4 experiments (error bars indicate SEM).

We found that, while DSB repair timing is unchanged, JM levels are substantially reduced during IS repair. Maximum JM frequencies at *his4::URA3-arg4* were reduced 2-fold in hemizygote or left arm deletion strains relative to homozygous controls (1.2%±0.2% versus 2.4%±0.2%; Figure 3A). Similarly, JMs at *YCR047c* were reduced about 4-fold in *YCR047c* hemizygotes as compared to homozygous controls (0.5%±0.1% versus 2.0%±0.1%; Figure 3A).

Reduced steady-state levels of JMs can result either from reduced JM formation or from decreased JM life span. To distinguish between these possibilities, JM levels were measured in resolution-defective *ndt80*Δ strains. Cumulative JM frequencies at a hemizygous *his4::URA3-arg4* insert were about 2-fold lower than in homozygous controls (4.6%±0.5% versus 8.8%±0.6%; Figure 3B and 3C), where repair can occur from either the sister or the homolog. JM frequencies at *YCR047c* were similarly reduced in *ndt80*Δ hemizygotes relative to homozygous controls (2.6%±0.6% versus 5.9%±1.2%; Figure 3B and 3C). The approximately 2-fold decrease in both cumulative and steady-state JM frequencies is consistent with the suggestion that JM formation, rather than life span, is reduced during the repair of DSBs that form in a region of short insertion/deletion heterology. This, in turn, indicates that meiotic DSB repair events by IS recombination, when the sister is the only template for repair, produces a lower fraction of JMs than DSB repair when both homolog and sister are present, and the majority of JMs form between homologs. These results suggest that previous estimates of the relative levels of IS and IH recombination, which were based on JM levels [7],[10]–[12], may have underestimated the fraction of recombination that occurs between sister chromatids (see Discussion).

In contrast, *ndt80*Δ strains hemizygous for the 90-kb left arm deletion accumulated JMs at *his4::URA3-arg4* in two phases. At earlier times (up to 4.5 h, when JMs begin to disappear with wild-type), JMs were present at frequencies similar to those in strains with the much shorter *his4::URA3-arg4* heterology. At later time points, JMs accumulated much more rapidly, reaching JM levels seen in homozygous control strains (Figure 3C). These results suggest that the outcome of IS recombination can be influenced by IH interactions in flanking chromosomal regions (see Discussion).

### Msh4 Is Required for Wild-Type Levels of IS Joint Molecules

Several meiosis-specific proteins, collectively called the ZMM proteins, are required for wild-type levels of JMs and COs and normal synaptonemal complex formation, but not for normal NCO levels [37]–[39]. Two of these, Msh4 and Msh5, form a heterodimer that is thought to promote JM formation by stabilizing early recombination intermediates [37],[40],[41]. IS and IH JM formation are reduced in *msh5* strains [11],[38], but it has not been determined whether IH and IS JMs are equally affected. We therefore measured cumulative JM levels in *msh4*Δ *ndt80*Δ strains that were hemizygous or homozygous for the *his4::URA3-arg4* insert. A 3-fold decrease in both IS and total JM frequencies was observed in both the hemizygous and homozygous *msh4*Δ *ndt80*Δ strains (Figure 3D). Assuming that the majority of JMs in homozygous *msh4*Δ *ndt80*Δ strains are IH JMs, it can be concluded that IS and IH JMs are similarly *MSH4*-dependent.

### Altered DSB Repair and JM Metabolism in *mek1*Δ Cells

**Figure 4:**
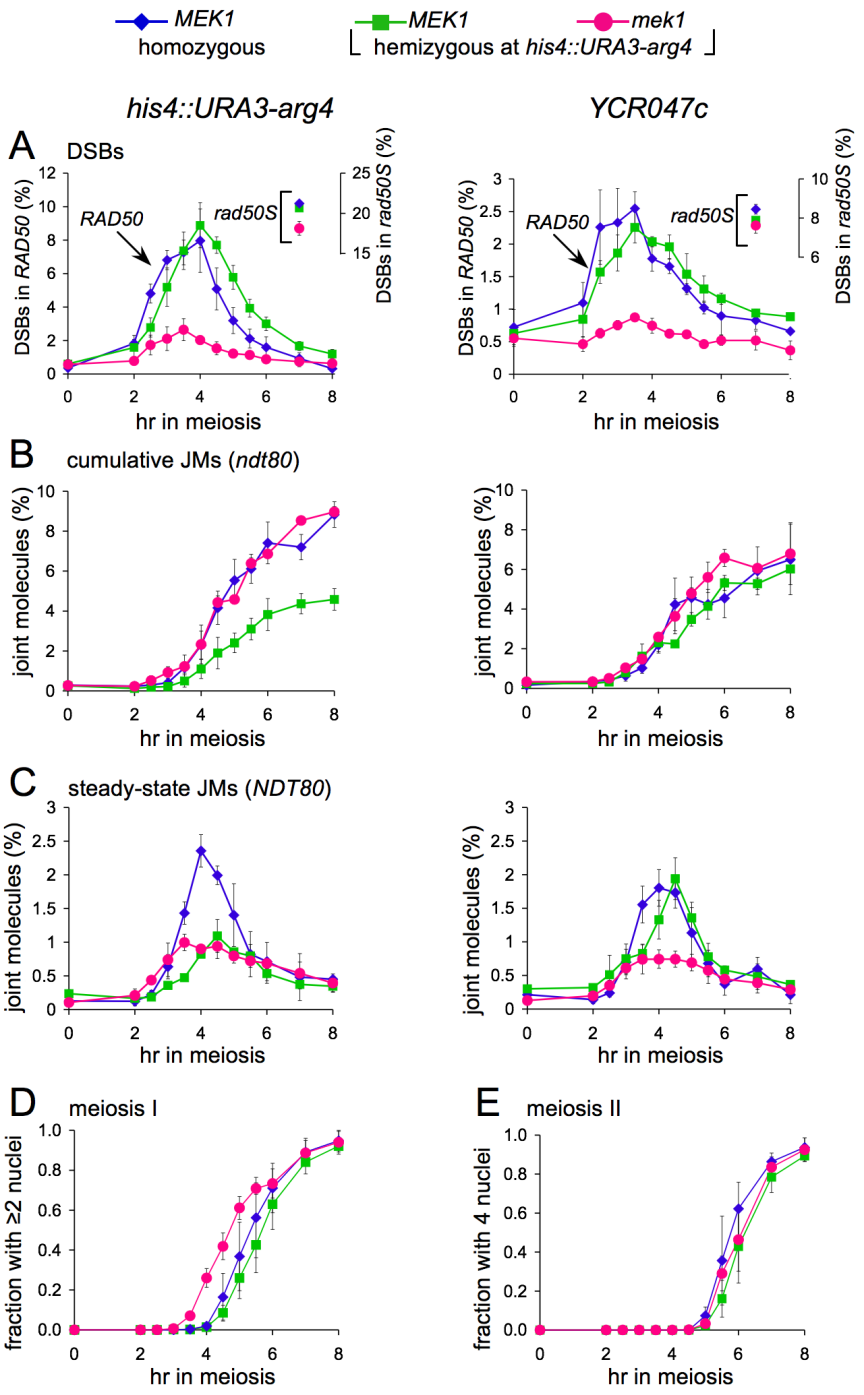
Altered DSB and JM metabolism in *mek1*Δ strains. (A) DSB frequencies (3–4 independent experiments, error bars indicate SEM), quantified as percent of total lane signal. Symbols: blue diamonds, fully homozygous *MEK1* strain (MJL3201 and MJL3198); green squares, *MEK1* strains hemizygous for *his4::URA3-arg4* (MJL3250 and MJL3338); and pink circles, *mek1*Δ strain hemizygous for *his4::URA3-arg4* (MJL3370 and MJL3381). Symbols connected by lines are noncumulative DSB frequencies from *RAD50* strains (left-hand *y*-axis; MJL3201, MJL3250, and MJL3370); unconnected symbols at 7 h are cumulative DSB frequencies from *rad50S* strains (right-hand *y*-axis; MJL3198, MJL3338, and MJL3381). (B) Cumulative JM frequencies (3–4 independent experiments, error bars indicate SEM) from *ndt80*Δ *MEK1* and *ndt80*Δ *mek1*Δ strains. Symbols: blue diamonds, fully homozygous *MEK1* strain (MJL3252); green squares, *MEK1* strains hemizygous for *his4::URA3-arg4* (MJL3497); and pink circles, *mek1*Δ strain hemizygous for *his4::URA3-arg4* (MJL3387). (C) Noncumulative JM frequencies (3–4 independent experiments, error bars indicate SEM) from the same *RAD50* strains used for DSB analysis in (A). (D and E) Timing of the meiosis I (D) and meiosis II (E) nuclear divisions, monitored by DAPI staining (see Protocol S1; 3–4 independent experiments, error bars indicate standard deviation), in the same *RAD50* strains used for DSB and JM analysis in (A) and (C).

DSBs form at normal levels but are more rapidly repaired in strains lacking Mek1 kinase activity, as compared to wild-type [17],[19],[21]–[23],[42]. A similar decrease in DSB life span is seen in cells with an unphosphorylatable Hop1 protein that does not activate the Mek1 kinase [18]. Because *mek1* strains also show greatly reduced IH recombination [21],[23],[42],[43], it has been suggested that, in the absence of Mek1 activity, meiotic DSBs are rapidly repaired by IS recombination. We confirmed that, in *mek1*Δ strains, steady-state DSB levels are substantially reduced at a hemizygous *his4::URA3-arg4* insert and at a homozygous *YCR047c* site, while cumulative DSB levels, measured in *rad50S* strains, are not affected (Figure 4A). Thus, DSB life spans are substantially reduced in *mek1*Δ relative to wild-type (by about 3-fold; data not shown). Because DSBs in hemizygous loci are repaired by IS recombination, this indicates that loss of Mek1 increases the rate of IS recombination by about a factor of three.

In addition to accelerating IS recombination, loss of Mek1 alters the fraction of IS events that form JMs. In contrast to the 2-fold reduction in IS JMs observed at hemizygous loci in *MEK1 ndt80*Δ strains, IS JM levels at hemizygous loci in *mek1*Δ *ndt80*Δ strains were similar to those observed for homozygous loci in *MEK1 ndt80*Δ strains, where most JMs are IH (Figure 4B, left panel). Thus, the Mek1 kinase decreases the rate and alters the outcome of IS recombination.

In contrast, steady-state JM levels in *mek1*Δ *NDT80* cells are about 2- to 2.5-fold reduced, relative to wild-type, at both hemizygous and homozygous loci (Figure 4C). Since cumulative JM levels are unreduced in *mek1*Δ *ndt80*Δ cells, this indicates that JM life spans are shortened in *mek1*Δ. This may be due to accelerated meiotic progression caused by the early loss of DSB signal, since *mek1*Δ cells undergo the first meiotic nuclear division about 40 min earlier than do *MEK1* cells (Figure 4D; [42]), as do DSB-defective mutants [42],[44]–[46]. This early MI division most likely results from early activation of the *NDT80* transcriptional program [46], which is also responsible for JM resolution [8],[47]. Ndt80 is a target of the meiotic DNA damage response [48], and reduced steady-state DSB levels in *mek1*Δ may, in turn, lead to reduced DNA damage signaling and thus premature activation of Ndt80.

**Figure 5:**
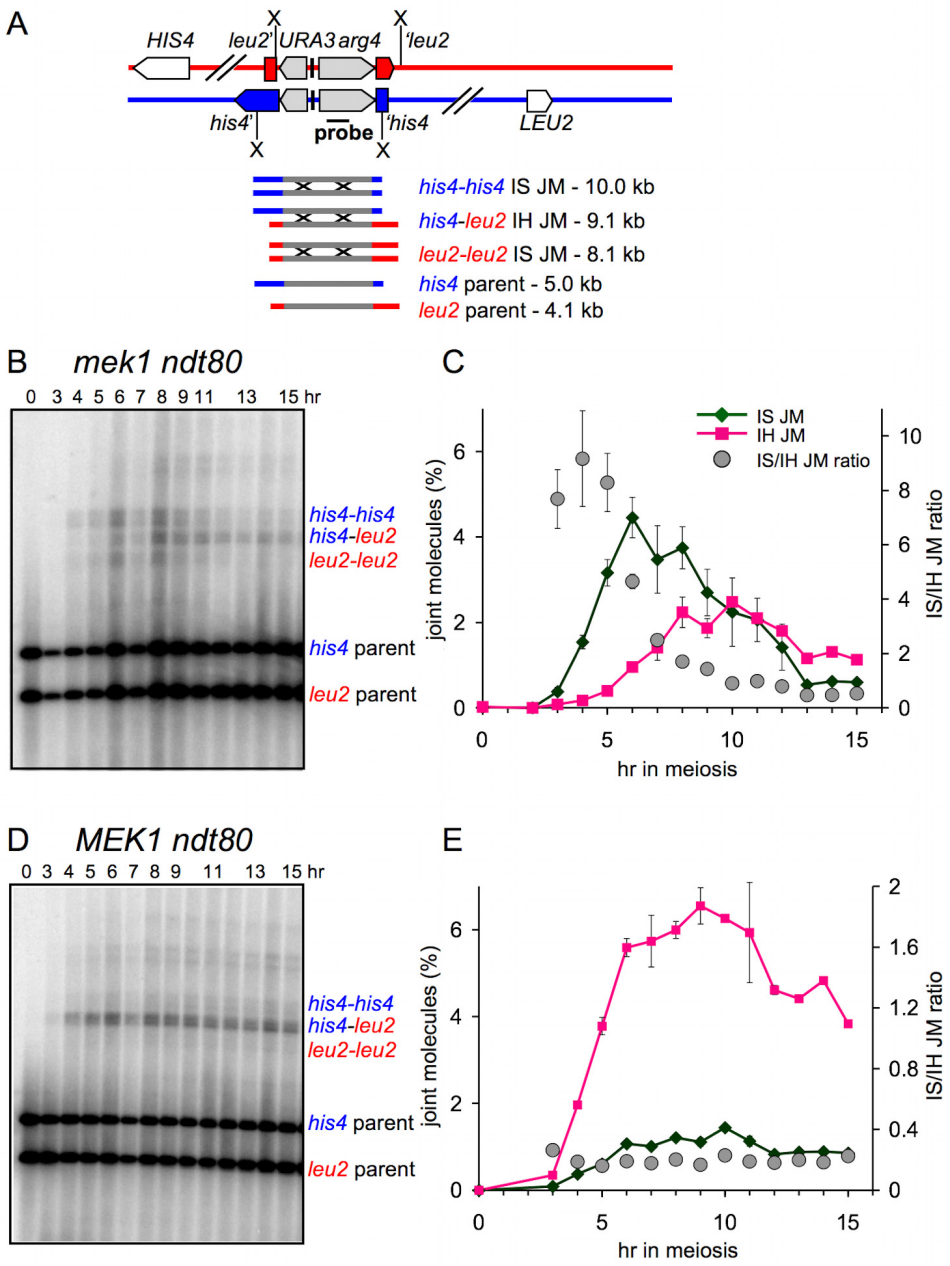
IS JM formation in *mek1*Δ. (A) Recombination assay system used to distinguish IS and IH JMs. The *URA3-arg4* construct is inserted at *LEU2* on one homolog (red) and at *HIS4* on the other homolog (blue). Digestion with XmnI (X) produces IS JMs and IH JMs that can be distinguished on the basis of electrophoretic mobility. (B) Representative Southern blot of DNA from a *mek1*Δ *ndt80*Δ strain with *his4::URA3-arg4* and *leu2::URA3-arg4* insert (MJL3397). (C) Frequencies (left *y*-axis, percent of total lane signal, three independent experiments, error bars indicate SEM) of IH JMs (pink squares, *his4*-*leu2* band in [B]) and IS JMs (green diamonds, sum of *his4*-*his4* and *leu2*-*leu2* bands in [B]). Grey circles: IS/IH JM ratio (right *y*-axis). (D) Representative Southern blot of DNA from a *MEK1 ndt80*Δ strain with *his4::URA3-arg4* and *leu2::URA3-arg4* insert (MJL3523). (E) Frequencies (left *y*-axis, percent of total lane signal, two independent experiments, error bars indicate SEM) of IH JMs (pink squares, *his4*-*leu2* band in [D]) and IS JMs (green diamonds, sum of *his4*-*his4* and *leu2*-*leu2* bands in [D]). Grey circles: IS/IH JM ratio (right *y*-axis).

IH recombination levels are markedly reduced in *mek1* mutants [21],[23],[42],[43], suggesting that even when IH recombination is possible, IS repair predominates in *mek1*Δ mutants. To confirm this, we examined *ndt80*Δ strains where JMs formed by IS and IH recombination in the *URA3-arg4* interval can be distinguished (Figure 5A; [29]). In *MEK1 ndt80*Δ strains, the majority of JMs at this locus (∼80%) formed between homologs, and the IH/IS JM ratio was relatively invariant over time (Figure 5D and 5E; [12],[47]). In *mek1*Δ *ndt80*Δ, JMs accumulated to levels approaching those seen in *MEK1 ndt80*Δ, but most of the JMs initially formed in *mek1*Δ *ndt80*Δ were between sister chromatids (IS/IH JM ratio of ∼8∶1 for the time interval 3–5 h after transfer to sporulation medium; Figure 5B–5E). With continued incubation in the *ndt80*Δ-arrested state (6 h and later), IS JM levels decreased and IH JM levels increased. IH JMs roughly equaled IS JMs by 10–12 h after transfer to sporulation medium and became the majority class by 13 h (Figure 5B and 5C), although maximum IH JM frequencies (2%–2.5%) were much less than those seen in *ndt80*Δ *MEK1* (6%–7%).

Thus, in contrast to what is observed in the presence of Mek1, IS recombination predominates during initial JM formation in the absence of Mek1, a finding also reported by Kim and coworkers [24]. In addition, the differential loss of IS JMs at later times is consistent with the suggestion that IS JMs are less stable than are IH JMs [11],[12],[49],[50]. Thus, while Mek1 plays a major role in regulating IS recombination during meiosis, other activities impact the outcome of IS recombination in the absence of Mek1.

## Discussion

### The Sister Chromatid Is Used Efficiently during Meiotic Recombination

Most studies of meiotic recombination have focused on recombination between homologs, and less attention has been given to the potentially critical role for recombination between sister chromatids. For example, a substantial fraction of variation among human haplotypes consists of insertion/deletion polymorphisms that are greater than 500 nucleotides in length [51],[52]. One way to ensure the timely repair of DSBs that form in regions of heterozygosity for such insertion/deletions would be to have both the homolog and sister chromatid available as potential partners.

**Figure 6:**
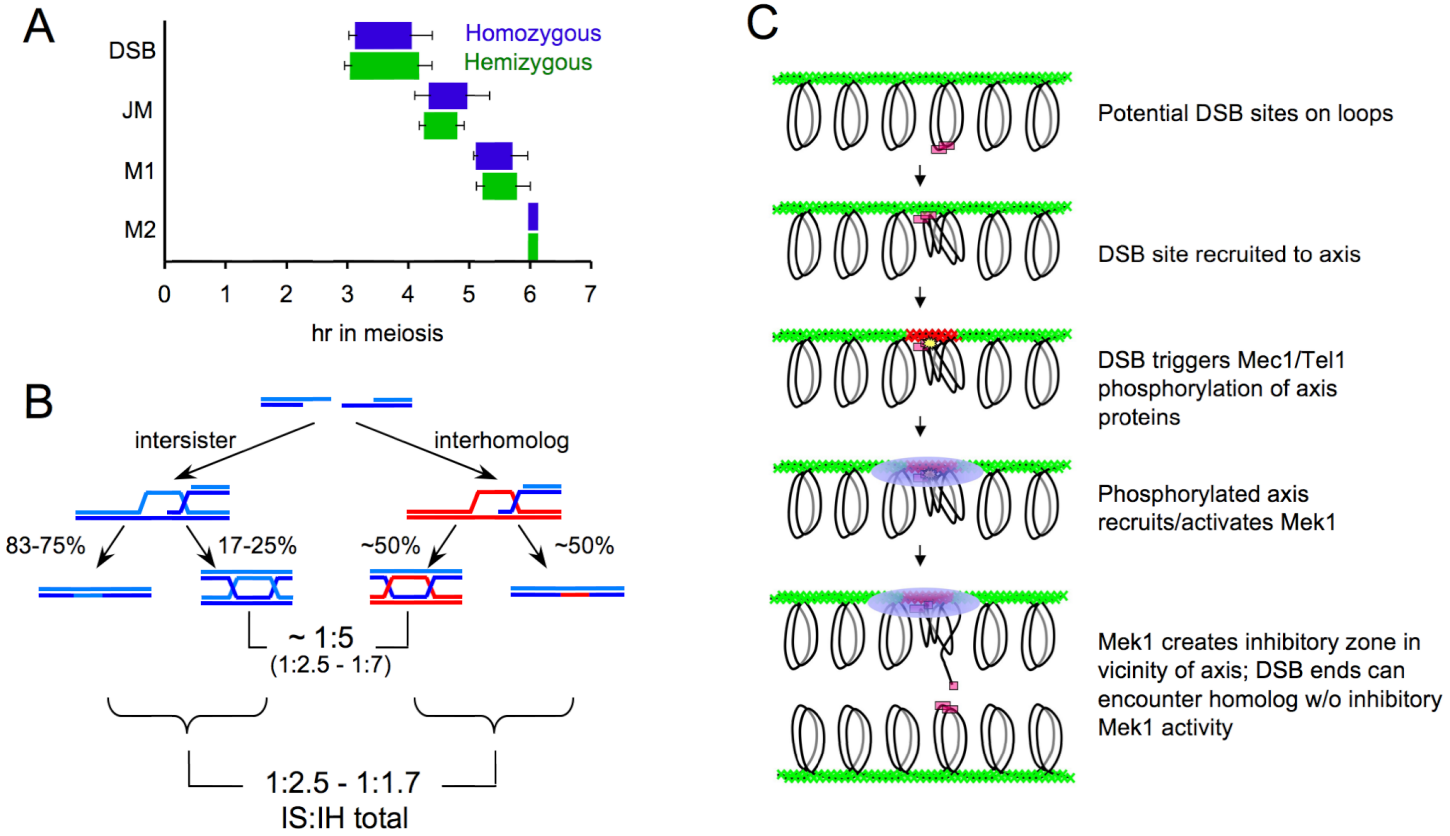
(A) Timing of molecular events at a hemizygous (MJL3250) and homozygous (MJL3201) *his4::URA3-arg4* insert. Left- and right-hand edges of rectangles indicate half-maximum points on cumulative curves for formation and repair/resolution, respectively. For meiotic divisions, left- and right-hand edges indicate 50% times for meiosis I (M1) and meiosis II (M2), respectively. Times are normalized by setting the 50% time for meiosis II to 6 h (actual times ± standard deviation: MJL3201, 5.66±0.27 h; MJL3250, 6.08±0.33 h). Left- and right-hand error bars denote the standard deviation for the half-maximum value and the sum of the standard deviations of the life span and the half-maximum value, respectively. (B) Estimation of IS/IH ratio for all recombination events at homozygous loci in wild-type, based on the following: (1) about 1/2 of IH events involve JMs; (2) about 1/4 to 1/6 of IS events involve JMs; (3) about 1/5 of JMs are IS (numbers in parentheses are observed range in the literature). Based on these values, an IS/IH total event ratio of 1∶1.7 to 1∶2.5 is calculated. Detailed calculations and IS/IH total event ratios for the full range of IS/IH JM ratios are in Protocol S1 and Figure S2. (C) How localized kinase activation can cause selective retardation of IS recombination. DSBs form when potential DSB sites on cohesed sister chromatids (pink boxes) are recruited to the chromosome axis (green). Mec1/Tel1 checkpoint kinases are activated by DSBs and associated single-stranded DNA, and phosphorylate chromatin and axis proteins in the vicinity of DSBs (red). Phosphorylated axis proteins recruit and activate Mek1 kinase, which phosphorylates target proteins (including strand transferase accessory proteins) in the vicinity of the DSB-activated axis. Strand invasion of the sister chromatid, which is within the zone of axis-associated inhibition, is thus kinetically impeded; strand invasion of the homolog is unaffected.

Our genetic and molecular data indicate that the sister chromatid can be used as efficiently as the homolog in the repair of meiotic DSBs. DSBs that form at hemizygous loci are repaired with the same efficiency and timing as DSBs formed at homozygous loci (Figures 1 and 6A). While these DSBs could, in theory, be repaired by IH gene conversion of the entire region of heterology, such events are relatively rare (Figure 2). We therefore conclude that the majority of DSBs that form at hemizygous loci are repaired by recombination between sister chromatids. Furthermore, the efficient repair of DSBs that form opposite deletions of an entire chromosome arm (Figure 1) indicates that nearby IH interactions are not required for IS recombination to occur. While repair in hemizygous strains occurs exclusively from the sister chromatid, Hunter and colleagues have suggested that multiple templates, including the sister chromatid, are frequently used in the repair of DSBs when both parental homologs are present [11]. JMs containing three and four chromatids form in wild-type cells, and are abundant in strains lacking the Sgs1 helicase [11]. This supports the suggestions that multiple repair templates are often used during meiotic recombination, that recombination is a dynamic process, and that Sgs1 acts to prevent aberrant structures that are formed as a result of these processes [11],[50]. The increased incidence of IS JMs in strains lacking Sgs1 further supports the claim that the sister chromatid is often used for DSB repair during meiosis [11],[12],[49].

The timely and efficient repair of DSBs at hemizygous loci contrasts with the pronounced DSB persistence and repair failure observed in haploid meiosis [13],[14] or in the absence of Dmc1 [53]. Efficient IS DSB repair is restored in *dmc1* diploids and haploid yeast when axis-dependent, DSB-dependent signaling through Mek1 is blocked [14],[17],[18],[21], and this has been taken as evidence for a Mek1-dependent barrier that prevents most DSBs from being repaired by IS recombination. Our finding and previous findings that IS and IH JMs appear with similar relative timing in budding yeast [10],[11],[38] are inconsistent with suggestions that most IS recombination occurs upon synaptic adjustment or axis breakdown late in meiosis I prophase. Rather, we suggest that IS recombination occurs with timing and frequencies similar to those of IH recombination. Our data suggest that in normal diploid meiosis, the constraint on IS strand invasion about equals the constraint on IH repair imposed by the need to search through the nucleus to find the homolog. Such an equalizing force would allow for the establishment of IH connections, while maintaining the ability to properly repair DSBs incurred in regions of heterozygosity. Possible explanations for why DSBs persist during meiosis in haploids and in *dmc1* mutants will be discussed below.

### Implications for Relative Levels of IS and IH Recombination

JM production at hemizygous loci is reduced 2- to 3-fold relative to total JM production when the same loci are homozygous (Figure 4). Since most JMs resolve as COs during meiosis [47], this would suggest that CO recombination is less prevalent, and NCO recombination is more prevalent, during IS recombination than during IH recombination. If this is true, then previous estimates of IS and IH recombination levels, based on the relative levels of IH and IS JMs [10]–[12],[38], would substantially underestimate the fraction of events that involve IS recombination (Figure 6B).

In estimating the total fraction of events that involve IS recombination, we consider that IH COs and NCOs are produced in roughly equal numbers at *his4*::*URA3*-*arg4* [29]. Since most COs are produced by JM resolution [8],[47], about one-half of all IH events at this locus involve JM formation. Since IS JMs levels at hemizygous loci are 2- to 3-fold lower (Figure 4), JMs constitute between one-sixth and one-quarter of IS recombination events at the hemizygous locus. If the same ratio holds for the IS recombination at homozygous loci, then previously reported IH/IS JM ratio (between 1∶2.5 and 1∶7; [24] can be used to estimate the fraction of IS events that involve NCO recombination, and thus are not detected (Figure 6B; Protocol S1). Using a consensus IH/IS JM ratio of 1∶5, the calculated ratio of IS/IH recombination is between 1∶1.7 (if IS JMs are reduced 3-fold relative to IH JMs) and 1∶2.5 (if IS JMs are reduced 2-fold). Thus, if our findings regarding IS recombination at hemizygous loci hold for IS recombination at homozygous loci, roughly one-third of meiotic DSBs may be repaired by IS recombination in budding yeast, a fraction expected on the basis of target copy number alone. It should be noted that this analysis assumes that the outcome of IS recombination is the same regardless of the presence or absence of corresponding sequences on the homolog, a fact that remains to be determined.

In addition, the calculated value of one-third is highly dependent on the actual IS/IH ratio of JMs formed during meiosis. While a consensus value of 1∶5 was used in this calculation, large variation in this value has been reported [10]–[12],[24]. Such variation would change estimates of the fraction of breaks repaired by the sister, accordingly (see Protocol S1). A true test of the predicted frequency of IS repair will require an accurate inventory of DSBs, COs, and NCOs at multiple individual loci, as well as genome-wide.

Despite the general impression that IH recombination predominates during meiosis, existing data indicate that IS recombination may be prevalent in other organisms. High levels of IS recombination have been documented at the fission yeast *mbs1* locus, where about 80% of meiotic JMs detected are between sister chromatids [54], and where DSBs are efficiently repaired during haploid meiosis [55]. About 20% of all COs detected by BrdU/FPG staining of locust spermatocytes are IS [56]. During mammalian meiosis, Rad51/Dmc1 foci (thought to mark DSBs) outnumber Mlh1 foci (thought to mark COs) by about 10- to 20-fold [57],[58], but sperm-typing studies measure NCO/CO ratios in the range of 3∶1 to 9∶1 [59]–[61]. While this may reflect a systematic underscoring of NCO events, it is also possible that a substantial fraction of meiotic DSB repair in mammals might occur by IS recombination.

### Mek1 Slows Down, but Does Not Block, IS Recombination

While previous studies have implicated the Mek1 kinase in reducing the frequency of IS events, our data provide the first quantitative measure, to our knowledge, of the extent to which Mek1 activity impairs IS recombination. DSB life spans at a hemizygous *his4::URA3-arg4* locus are reduced 3-fold in *mek1*Δ mutants relative to *MEK1* (Figure 4). This would suggest that Mek1 imposes a 3-fold reduction in the rate of IS repair. Our observation of similar rates of DSB repair at hemizygous and homozygous loci (Figure 1) suggests that Mek1 reduces the rate of IS strand invasion to the point where it is similar to the overall rate of strand invasion of the homolog, a process where the homology search is probably the rate-limiting step [62],[63]. It is of interest to note in this context that the *rad52-Y66A* allele, which substantially slows mitotic DNA damage repair, substantially increases the frequency of mitotic IH recombination [64].

In *MEK1 ndt80*Δ strains, IS JMs at hemizygous loci are reduced 2- to 3-fold relative to JMs formed when both homologs are present, but no such reduction is seen in *mek1*Δ *ndt80*Δ (Figure 4). This Mek1-dependent reduction in JM formation may simply be due to Mek1's effect on IS strand invasion. If Mek1 impairs strand invasion, such activity could substantially reduce JM production, since the formation of such intermediates requires two separate strand invasion events. Alternatively, it may reflect interference caused by nearby events that form IH JMs. If interference acts both on events that form IH JMs and on events that form IS JMs, the lack of IH JMs in *mek1*Δ would lead to elevated IS JM production. Consistent with this latter suggestion, elevated IS JM levels are also seen at later times in *MEK1 ndt80*Δ strains where *his4::URA3-arg4* is opposite a 90-kb deletion on the chromosome *III* left arm, precluding the possibility of nearby IH events (Figure 4). Further studies are needed to test this possibility.

### Dual Mechanisms Promote IH Recombination during Meiosis

We have shown here that the presence of the Mek1 kinase imposes a kinetic constraint on IS recombination. Slowing this process, which otherwise would be rapid because of the close proximity of the sister chromatid, allows time for the genome-wide homology search needed for DSBs to engage in IH recombination, while retaining the ability to efficiently repair DSBs if homology is not encountered. Thus, the extent of reduction in IS recombination would be expected to vary among organisms, depending upon the nature of the search necessary for IH recombination. IS recombination might be minimally constrained in *Schizosaccharomyces pombe*, where homologs are extensively co-localized and co-aligned at the time of DSB formation [55],[65], while substantial kinetic constraints might be imposed in organisms with large and complex genomes.

If a kinetic constraint on strand invasion is to have a differential effect on IS and IH recombination, additional specificity must be involved. One way to accomplish this would be to target proteins specialized for IS recombination, as has been suggested for the Rad51/Rad54 combination [66]. However, this suggestion is challenged by the observation that increasing Rad51 protein levels or activity can partially suppress the IH recombination defect of yeast *dmc1* mutants [20],[25],[26],[67],[68], and by the existence of organisms where Rad51 is the sole source of meiotic strand transfer activity [69]–[72]. The existence of documented anti-recombination activities conferred by the Hed1 protein and by the Mek1-dependent phosphorylation of Rad54 and Rdh54 [20],[68],[73] supports the idea that strand invasion activities are constrained. However, for such anti-recombination activities to be specific to the sister, Mek1-mediated anti-recombination activity must be spatially restricted to chromosomal regions near DSBs, thus locally inhibiting sister chromatid recombination while allowing unconstrained DSB end-invasion of the homolog (Figure 6C; [19],[50]). This suggestion is based on the observation that DSB-induced, Mec1/Tel1 chromatin modification occurs primarily in a gradient within a 50- to 100-kb region around the break [74], and on the hypothesis that activated Mek1 kinase has a relatively short half-life. This idea of a spatially confined Mek1 activity is distinct from what is observed for activated Rad53, whose release from Rad9 leads to amplification of the checkpoint signal throughout the cell [75]. Under normal circumstances, when DSBs are being formed and repaired asynchronously [76], a Mek1-dependent zone of recombination inhibition will primarily involve the broken chromosome and its sister chromatid, leading to a differential slowing of IS recombination. However, under conditions where DSB repair is delayed (such as in *dmc1* mutants, or when all homologs are absent), there is the possibility that increased overall levels of single-stranded DNA will lead to elevated Mec1/Tel1 signaling and consequent hyperactivation of Mek1, thus transforming a localized kinetic constraint into the observed genome-wide inhibition of recombination. Experiments to test this suggestion are ongoing.

Once established, IH connections must be maintained, so that they can be resolved as the COs necessary for proper homolog segregation at the first meiotic division. Our data are also consistent with suggestions that differential stabilization of IH JMs may contribute to their preponderance over IS JMs [50]. *mek1*Δ *ndt80*Δ strains display an excess of IS JMs at early time points (Figure 5), but over time IS JMs decrease and IH JMs increase, suggesting that IH JMs are preferentially stabilized, IS JMs are preferentially destabilized, or both. Our current data do not distinguish between these alternatives, and do not address the issue of whether or not similar mechanisms operate in *MEK1* cells, where the Sgs1 helicase has been implicated in reducing formation of JMs that contain IS interactions in wild-type [11],[37]. It will be of considerable interest to examine the impact of Sgs1 on JM formation at hemizygous loci, and examine the possible role of Mek1-mediated phosphorylation in recruiting potential JM-destabilizing activities.

## Materials and Methods

### Strains

All yeast strains are derived from SK1 [77]. See Protocol S1 and Table S1 for genotypes and construction. The 3.5-kb *URA3*-*arg4* insert has been previously described [29]. The chromosome *III* left arm deletion replaces 90 kb between *YCL069w* and *YCL004w* with the 1.5-kb hygromycin resistance cassette *hphMX4* [78]. The *YCR047c* deletion replaces 4 kb between *YCR046c* and the middle of *YCR051w* with *hphMX4*.

### Sporulation and Genomic DNA Preparation

Sporulation in liquid, DNA extraction, and recombination product and intermediate analysis were as described previously [37],[79],[80] with modifications. DSB life span and cumulative curves for DSB formation and repair were calculated as described previously [81]. Nuclear divisions were monitored by DAPI staining. Details are in Protocol S1.

## Supporting Information

Protocol S1 **Supplementary methods and references.**
(0.17 MB PDF)

Figure S1 **Calculated DSB formation and repair curves.** (A) Structure of strains used to study meiotic recombination. Symbols as in Figure 1. (B) Calculated curves of DSB formation for DSBs at the indicated locus. Noncumulative DSB values (Figure 1C) were converted to cumulative curves as described previously [81]. Error bars indicate standard error of the mean (SEM). (C) DSB repair curves, obtained by shifting DSB formation curves rightward by calculated DSB life span values (Figure 1D). (D) Relative DSB levels at three independent loci (*his4::URA3-arg4*, *YCR047c*, and *YFL021w*) from a single experiment with a strain (MJL3399) hemizygous for *YCR047c*.
(0.32 MB PDF)

Figure S2 **Calculation of fraction of all events involving IS recombination.** (A) Logic of calculation. See Protocol S1 for details. (B) IS events/total events ratios were calculated as described in Protocol S1, for three values of *j*, the fraction of IS events that form JMs: open circles indicate that the fraction of IS events forming JMs is 2-fold reduced relative to the fraction of IH events forming JMs; closed circles indicate that the fraction of IS events forming JMs is 3-fold reduced relative to the fraction of IH events forming JMs. The dotted line indicates the ratio of IS events/total events expected if the same fraction of IS and IH events form JMs.
(0.10 MB PDF)

Table S1 **Strains used.** All are SK1 *MAT* **a**/*MAT*α diploids and are homozygous for *ura3*Δ*(HindIII-SmaI) lys2 ho*::*LYS2 arg4*Δ*(Eco47III-Hpa1)*.
(0.08 MB PDF)

## Abbreviations

BSCR: barrier to sister chromatid recombination
CO: crossover
DSB: double-strand break
IH: inter-homolog
IS: inter-sister
JM: joint molecule
MI: first meiotic division
NCO: noncrossover
SEM: standard error of the mean
