# Supplementary material for: Frequent and Efficient Use of the Sister Chromatid for DNA Double-Strand Break Repair during Budding Yeast Meiosis
Source: PLoS Biol. 2010 Oct 19;8(10):e1000520. doi: 10.1371/journal.pbio.1000520 (PMC2957403; doi:10.1371/journal.pbio.1000520)
Supplement: Protocol S1 — Supplementary methods and references. (0.17 MB PDF) [file pbio.1000520.s001.pdf]

Protocol S1. Supplementary Methods and References

Strains. All strains (Supplementary Table S1) were derived from SK1 by transformation or genetic crosses. Chromosome *III* left arm deletion and *YCR047c* deletion strains were generated by transforming a diploid strain with a PCR-generated fragment containing the 1.5 kb hygromycin B phosphotransferase gene (*hphMX4*)[1] flanked by homology to chromosomal sequences at the deletion endpoints (primer sequences provided upon request). The chromosome *III* left arm deletion starts 286 bp downstream of the *YCL069w* start site and ends 60 bp downstream of the *YCL005W* start site, and was verified by pulsed-field gel electrophoresis and PCR analysis (data not shown). In strains hemizygous for the *YCR047c* deletion, *hphMX4* replaces 4 kb of chromosome III sequences, on the *MAT***a** chromosome, beginning 1 bp downstream of the *YCR047c* stop codon and extending to the +657 position in the *YCR051w* ORF, and was verified by PCR analysis (data not shown).

Sporulation and genomic DNA preparation. Liquid sporulation of cultures and DNA isolation was as described [2,3], with minor modifications. 1% potassium acetate sporulation medium supplemented with 0.001% polypropylene 2000 (Aldrich), 6 μg of lysine per ml, 10 μg of arginine per ml, and 10 μg of histidine per ml. At each time point, 30 ml samples were collected and added to 8 ml of ice cold 50% glycerol. Samples were centrifuged at 4 °C and washed with 1 ml of ice cold 1.2 M sorbitol, 50 mM potassium phosphate (pH 7), 10 mM EDTA (pH 8), 20% glycerol. Cell pellets were frozen on dry ice and DNA extraction was performed as described [3], except that β-mercaptoethanol was omitted from the extraction buffer added to spheroplasted cells.

Meiotic nuclear divisions were monitored as follows. Meiotic cultures were collected and fixed in a final concentration of 50% ethanol. Samples were centrifuged, washed with water,

and resuspended with ProLong Gold with 4',6-diamidino-2-phenylindole (Molecular Probes). At least 200 cells were scored at each time point. Cells with either two DAPI staining bodies or a stretched nucleus were scored as having undergone an MI division. Cells with 4 DAPI staining bodies or containing 2 stretched nuclei were scored as having undergone the MII division.

Molecular analysis. To visualize DSBs at either the *his4::URA3-arg4* insert or *YCR047c*, 0.5-1 μg genomic DNA was digested with either *Xho*I (50 units - Fermentas) or *Bgl*II (40 units – Roche), respectively, in the recommended buffer supplemented with 0.1 mg/ml bovine serum albumin (BSA). 1% agarose gels were run in 1X TAE (Sambrook et al., 1989), depurinated in 0.25 N HCl for 20 minutes, treated for 20 minutes in denaturing buffer (1.5 M NaCl, 0.5 N NaOH) and transferred to Hybond XL membrane (GE Healthcare) by either capillary or vacuum blotting in denaturing buffer. For JM analysis at either *URA3-arg4* or *YCR047c*, 0.5 - 1 μg genomic DNA was digested with either *Xmn*I (20 units – New England Biolabs) or *Hind*III (50 units – Fermentas), respectively, in the recommended buffer supplemented with 0.1 mg/ml bovine serum albumin (BSA) and 0.1 mM spermidine. Sample buffer containing a final concentration of 5 mM $MgCl_2$ was used, and gels (0.5 % agarose, 1X TBE, 4 mM $MgCl_2$) were run with recirculating buffer (1X TBE, 3 mM $MgCl_2$). Gels were washed 4 times (15 minutes each), twice with 4 gel volumes of 10 mM EDTA (pH 8.0), and then another two times in 5 mM EDTA (pH 8.0). Gels were depurinated and dentatured as described above, transferred by vacuum transfer to HybondXL membrane (GE Healthcare), and hybridized as recommended by the manufacturer. Probe sequences for *ARG4* (arg4D) were as described [4], and the *YCR051w* probe was a PCR fragment consisting of nucleotides 10 to 660 relative to the *YCR051w* translation start site. Probes were ^{32}P labeled using the HighPrime labeling kit as instructed by the manufacturer (Roche). Radioactive signal on filters was detected using a Fujifilm FLA-5100

phosphorimager and quantified using ImageGuage v4.22 software. DSB life spans were calculated by dividing the area under the steady-state DSB curve by the cumulative level of DSBs at that locus [5], and cumulative curves of DSB entry and exit were calculated using steady-state DSB levels and DSB life spans as described [5].

Calculation of IS/IH recombination ratio. Logic of this calculation is illustrated in Figure S2. The following assumptions and observations were used:

1. JM levels measured in *ndt80*Δ mutants reflect the total cumulative levels of JMs formed in wild-type.
2. About ½ of all IH recombination events form crossovers (Jessop and Lichten, 2005), and thus proceed via JM intermediates (Allers and Lichten, 2001; Börner et al., 2004).
3. The frequency of total events (in terms of events per locus) is similar at hemizygous and homozygous loci. This assumption is supported by the observation that DSB frequencies are similar at hemizygous and homozygous loci (Figure 1).
4. The fraction of IS recombination events involving JM formation is similar at hemizygous and homozygous loci.

Let ***f*** denote the frequency of DSBs at a locus. For a homozygous locus, let **s** denote the fraction of events involving intersister recombination, and ***r*** denote the IS-JM/IH-JM. For a hemizygous locus, let ***j*** denote the fraction of event that form JMs. If ***j*** is the same for IS recombination at hemizygous and homozygous loci, then

$$\frac{f \times j}{f} = \frac{f(1-s) \times r/2}{f \times s} \quad \text{or} \quad j = \frac{(1-s) \times r/2}{s}$$

solving for ***s***

$$s = \frac{r}{2j + r}$$

The observed reduction in IS JMs relative to IH JMs that we observe (2 to 3-fold, Figure 3) corresponds to ***j*** values of 0.25 and 0.17, respectively. If the same fraction of IS events form JMs as do IH events (which is not observed), then ***j*** = 0.5. The fraction of events calculated to be IS (***s***) are plotted as a function of IH-JM/IS-JM ratios (***r***) for these three values of ***j*** in Figure S2b.

References

1. Goldstein AL, McCusker JH (1999) Three new dominant drug resistance cassettes for gene disruption in *Saccharomyces cerevisiae*. Yeast 15: 1541-1553.

2. Goyon C, Lichten M (1993) Timing of molecular events in meiosis in *Saccharomyces cerevisiae*: stable heteroduplex DNA is formed late in meiotic prophase. Mol Cell Biol 13: 373-382.

3. Allers T, Lichten M (2000) A method for preparing genomic DNA that restrains branch migration of Holliday junctions. Nucleic Acids Res 28: e6.

4. Allers T, Lichten M (2001) Differential timing and control of noncrossover and crossover recombination during meiosis. Cell 106: 47-57.

5. Padmore R, Cao L, Kleckner N (1991) Temporal comparison of recombination and synaptonemal complex formation during meiosis in *S. cerevisiae*. Cell 66: 1239-1256.
