# Supplementary figures and images for: Frequent and Efficient Use of the Sister Chromatid for DNA Double-Strand Break Repair during Budding Yeast Meiosis
Source: PLoS Biol. 2010 Oct 19;8(10):e1000520. doi: 10.1371/journal.pbio.1000520 (PMC2957403; doi:10.1371/journal.pbio.1000520)

Goldfarb Figure S1

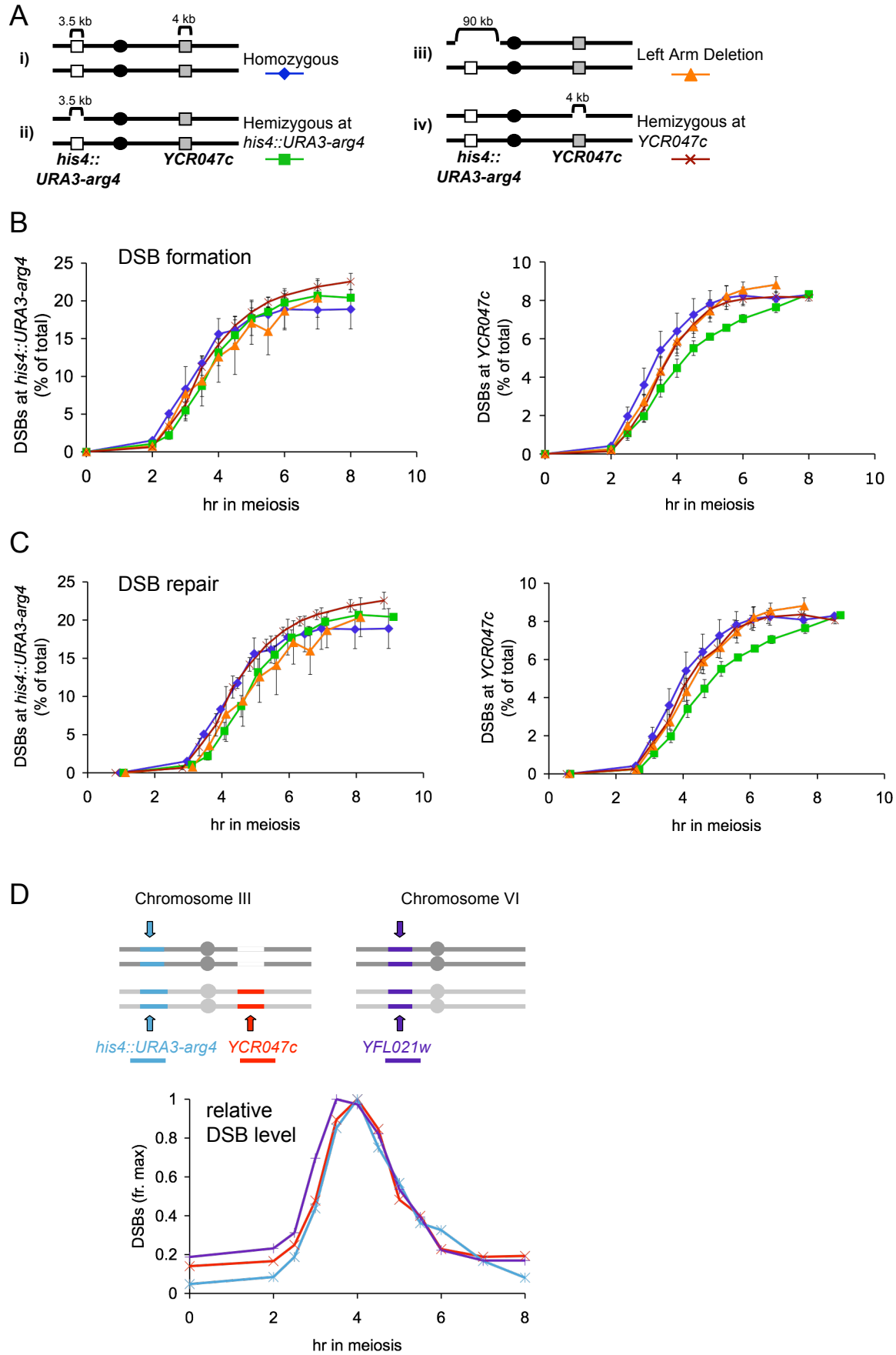

Supplement: Figure S1 — Calculated DSB formation and repair curves. (A) Structure of strains used to study meiotic recombination. Symbols as in Figure 1. (B) Calculated curves of DSB formation for DSBs at the indicated locus. Noncumulative DSB values (Figure 1C) were converted to cumulative curves as described previously [81]. Error bars indicate standard error of the mean (SEM). (C) DSB repair curves, obtained by shifting DSB formation curves rightward by calculated DSB life span values (Figure 1D). (D) Relative DSB levels at three independent loci (his4::URA3-arg4, YCR047c, and YFL021w) from a single experiment with a strain (MJL3399) hemizygous for YCR047c. (0.32 MB PDF) [file pbio.1000520.s002.pdf]

A

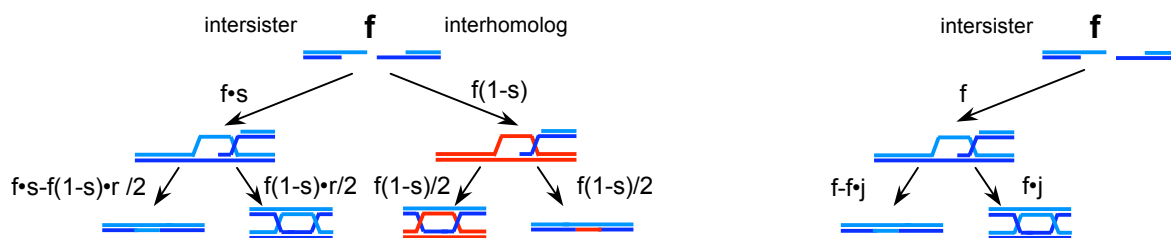

B

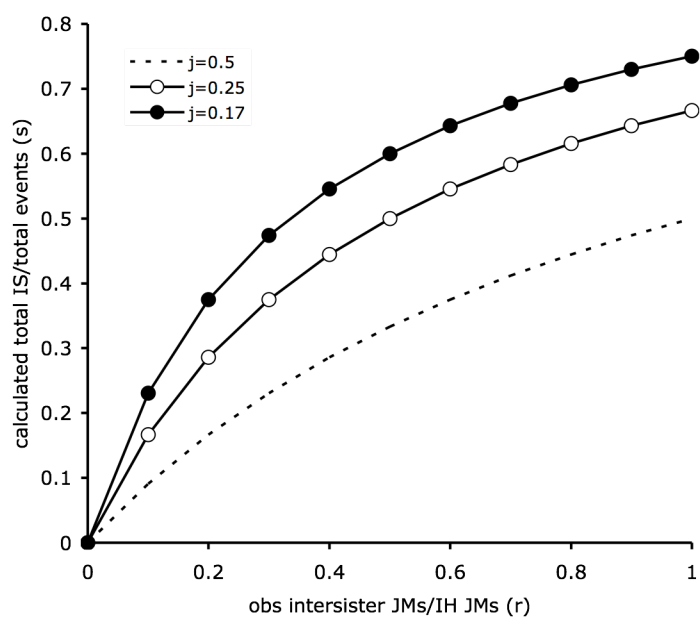

Supplement: Figure S2 — Calculation of fraction of all events involving IS recombination. (A) Logic of calculation. See Protocol S1 for details. (B) IS events/total events ratios were calculated as described in Protocol S1, for three values of j, the fraction of IS events that form JMs: open circles indicate that the fraction of IS events forming JMs is 2-fold reduced relative to the fraction of IH events forming JMs; closed circles indicate that the fraction of IS events forming JMs is 3-fold reduced relative to the fraction of IH events forming JMs. The dotted line indicates the ratio of IS events/total events expected if the same fraction of IS and IH events form JMs. (0.10 MB PDF) [file pbio.1000520.s003.pdf]
