# Supplementary material for: Frequent and Efficient Use of the Sister Chromatid for DNA Double-Strand Break Repair during Budding Yeast Meiosis
Source: PLoS Biol. 2010 Oct 19;8(10):e1000520. doi: 10.1371/journal.pbio.1000520 (PMC2957403; doi:10.1371/journal.pbio.1000520)
Supplement: Table S1 — Strains used. All are SK1 MAT a/MATα diploids and are homozygous for ura3Δ(HindIII-SmaI) lys2 ho::LYS2 arg4Δ(Eco47III-Hpa1). (0.08 MB PDF) [file pbio.1000520.s004.pdf]

Table S1

Strain	Relevant Genotype	
MJL3195		*his4Δ(Sal1-Cla1)::ura3rev-ecPal104-tel-ARG4*<br>--<br>*his4Δ(Sal1-Cla1)::URA3rev-tel-arg4-ecpal9*
MJL3192		*HIS4*<br>---<br>*his4Δ(Sal1-Cla1)::URA3rev-tel-arg4-ecpal9*
MJL3201		*his4Δ(Sal1-Cla1)::URA3rev-tel-arg4-ecpal9*<br>---<br>*his4Δ(Sal1-Cla1)::URA3rev-tel-arg4-ecpal9*
MJL3198	MJL3201+	*rad50-KI81::kanMX6*<br>------------------<br>*rad50-KI81::kanMX6*
MJL3252	MJL3201+	*ndt80Δ(Eco47III-BseRI)::kanMX6*<br>------------------------------<br>*ndt80Δ(Eco47III-BseRI)::kanMX6*
MJL3386	MJL3201+	*msh4Δ::kanMX ndt80Δ(Eco47III-BseRI)::kanMX6*<br>------------ ------------------------------<br>*msh4Δ::kanMX ndt80Δ(Eco47III-BseRI)::kanMX6*
MJL3250		*his4-x*<br>---<br>*his4Δ(Sal1-Cla1)::URA3rev-tel-arg4-ecpal9*
MJL3338	MJL3250+	*rad50-KI81::kanMX6*<br>------------------<br>*rad50-KI81::kanMX6*
MJL3497	MJL3250+	*ndt80Δ(Eco47III-BseRI)::kanMX6*<br>------------------------------<br>*ndt80Δ(Eco47III-BseRI)::kanMX6*
MJL3340	MJL3250+	*msh4Δ::kanMX*<br>------------<br>*msh4Δ::kanMX*
MJL3385	MJL3250+	*msh4Δ::kanMX ndt80Δ(Eco47III-BseRI)::kanMX6*<br>------------ ------------------------------<br>*msh4Δ::kanMX ndt80Δ(Eco47III-BseRI)::kanMX6*
MJL3370	MJL3250+	*mek1Δ::LEU2*<br>-----------<br>*mek1Δ::LEU2*
MJL3387	MJL3250+	*mek1Δ::LEU2 ndt80Δ(Eco47III-BseRI)::kanMX6*<br>----------- ------------------------------<br>*mek1Δ::LEU2 ndt80Δ(Eco47III-BseRI)::kanMX6*
MJL3227	MJL3201+	*chrIII Δ(9993-102118)::hphMX4*<br>---<br>*his4Δ(Sal1-Cla1)::URA3rev-tel-arg4-ecpal9*
MJL3233	MJL3227+	*rad50-KI81::kanMX6*<br>------------------<br>*rad50-KI81::kanMX6*

MJL3245	MJL3227+	*ndt80Δ(Eco47III-BseRI)::kanMX6*<br>------------------------------<br>*ndt80Δ(Eco47III-BseRI)::kanMX6*
MJL3399	MJL3201+	*Δycr047c-ycr051w::hphMX4*<br>------------------------<br>*YCR047c*
MJL3408	MJL3399+	*rad50-KI81::kanMX6*<br>------------------<br>*rad50-KI81::kanMX6*
MJL3406	MJL3399+	*ndt80Δ(Eco47III-BseRI)::kanMX6*<br>------------------------------<br>*ndt80Δ(Eco47III-BseRI)::kanMX6*
MJL3397		*HIS4*<br>---<br>*his4Δ(Sal1-Cla1)::URA3rev-tel-arg4-ecpal9*<br><br>*leu2::URA3rev-tel-arg4-ecpal9 mek1Δ::LEU2*<br>----------------------------- ----------<br>*LEU2* *mek1Δ::LEU2*<br><br>*ndt80Δ(Eco47III-BseRI)::kanMX6*<br>------------------------------<br>*ndt80Δ(Eco47III-BseRI)::kanMX6*
MJL3523		*HIS4*<br>---<br>*his4Δ(Sal1-Cla1)::URA3rev-tel-arg4-ecpal9*<br><br>*leu2::URA3rev-tel-arg4-ecpal9*<br>-----------------------------<br>*LEU2*<br><br>*ndt80Δ(Eco47III-BseRI)::kanMX6*<br>------------------------------<br>*ndt80Δ(Eco47III-BseRI)::kanMX6*

Table S1. Strains used. All are SK1 *MAT***a**/*MATα* diploids and are homozygous for *ura3Δ(HindIII-SmaI) lys2 ho*::*LYS2 arg4Δ(Eco47III-Hpa1)*.
